# Comparison of Shoulder and Elbow Biomechanical Characteristics in Left- and Right-Handed Youth Baseball Players

**DOI:** 10.3390/jcm14248638

**Published:** 2025-12-05

**Authors:** Hitoshi Shitara, Tsuyoshi Tajika, Tsuyoshi Ichinose, Tsuyoshi Sasaki, Noritaka Hamano, Masataka Kamiyama, Ryosuke Miyamoto, Kurumi Nakase, Fukuhisa Ino, Takuma Kachi, Yuhei Hatori, Koichiro Yanai, Atsushi Yamamoto, Kenji Takagishi, Hirotaka Chikuda

**Affiliations:** Department of Orthopedic Surgery, Graduate School of Medicine, Gunma University, 3-39-22 Showa-Machi, Maebashi 371-8511, Japan; tajika@gunma-u.ac.jp (T.T.); ichinose.ortho@gmail.com (T.I.); tsasaki@gunma-u.ac.jp (T.S.); noritakahamano@gmail.com (N.H.); m-kamiyama@gunma-u.ac.jp (M.K.); r-miyamoto@gunma-u.ac.jp (R.M.); walnut0523@gmail.com (K.N.); inofuku423@gmail.com (F.I.); takuman21@gmail.com (T.K.); y.hatori1989@gmail.com (Y.H.); koichiro3830@gmail.com (K.Y.); atsushiymmt@gmail.com (A.Y.); kenjitakagishi26@gmail.com (K.T.); chikuda-tky@umin.ac.jp (H.C.)

**Keywords:** diagnostic baseline, left-handed pitchers, baseball, injury risk assessment, youth baseball, range of motion, humeral torsion

## Abstract

**Background/Objectives:** This study investigated biomechanical differences between right-handed (RHPs) and left-handed (LHPs) youth baseball players by analyzing shoulder and elbow range of motion (ROM), muscle strength, and humeral torsion. Side-to-side asymmetries were also examined to identify potential handedness-related adaptations. **Methods:** This cross-sectional study included 2008 youth baseball players (1829 RHPs and 179 LHPs) aged 9–13 years; female players were excluded because of their small number, and only male participants were analyzed. Shoulder and elbow ROM, muscle strength, and humeral torsion were evaluated, with humeral torsion data collected from 1024 measurements (946 RHPs, 78 LHPs). Group differences were analyzed using the Mann–Whitney U and Wilcoxon Signed-Rank tests. Logistic regression analysis identified independent factors associated with being an LHP, while Pearson correlation analyses explored the relationships between humeral torsion and external/internal rotation. **Results:** LHPs exhibited significantly larger nondominant shoulder external rotation (*p* < 0.001), dominant internal rotation (*p* = 0.003), dominant shoulder horizontal adduction (*p* = 0.007), dominant elbow flexion (*p* = 0.006), and side-to-side prone internal rotation strength ratio (*p* < 0.001). LHPs also showed smaller dominant shoulder external rotation (*p* = 0.012), nondominant shoulder internal rotation (*p* = 0.001), nondominant horizontal adduction (*p* = 0.037), dominant prone external rotation strength (*p* = 0.002), and humeral torsion (*p* = 0.031). Humeral torsion differences correlated with external rotation in LHPs (r = 0.236) and internal rotation in RHPs (r = −0.153). Predictors of left-handedness included lower dominant shoulder external rotation (OR = 0.937) and higher dominant elbow flexion (OR = 1.410). **Conclusions:** This study provides novel insights into the normal functional characteristics of LHPs, an area that has been relatively underexplored. These findings serve as a basis for future studies on risk assessment, injury prevention, and performance optimization in youth baseball players.

## 1. Introduction

Throwing is a critical skill in baseball, but the repetitive motion involved can lead to anatomical changes in the shoulder [1]. Reports suggest that baseball players exhibit variations in range of motion (ROM), specifically increased external rotation (ER) and reduced internal rotation (IR) [1,2], in their dominant shoulder compared with their nondominant shoulder. Additionally, humeral torsion refers to the rotational alignment of the humerus, while humeral retrotorsion specifically describes the posterior rotation of the humeral head. Increased humeral retrotorsion is an adaptation observed in throwing athletes, allowing for greater ER while reducing anterior shoulder stress [2,3].

Proper shoulder and elbow biomechanics are essential for generating velocity while minimizing injury risk. The pitching motion demands a balance between mobility and stability, with ER facilitating energy storage and IR enabling forceful ball release [4]. However, excessive ER or IR deficits have been linked to shoulder injuries, while altered elbow mechanics contribute to valgus overload and ulnar collateral ligament injuries [5]. Understanding these biomechanical components is crucial for identifying handedness-related adaptations and improving injury prevention strategies.

While handedness has been widely studied in cognitive neuroscience and motor control research, its impact on sports biomechanics remains less understood. Left-handed players (LHPs) represent a minority in baseball, accounting for roughly 10% of players, yet they often have a strategic advantage due to the rarity of left-handed opponents and differences in ball movement patterns [6,7]. This relative scarcity suggests that LHPs may undergo distinct training loads and neuromuscular adaptations than their right-handed counterparts. Additionally, handedness has been linked to asymmetries in joint loading, movement coordination, and central nervous system control, which may contribute to differences in shoulder and elbow biomechanics [8,9,10,11]. These factors raise a key question: Do LHPs exhibit distinct anatomical adaptations due to their handedness, or do they follow similar patterns of ROM and strength development as right-handed players (RHPs)? Understanding these potential differences is essential for optimizing training and injury prevention strategies.

Despite the well-documented adaptations in RHPs, the biomechanical characteristics of LHPs remain poorly understood. Prior studies have reported differences in shoulder ROM and humeral torsion between RHPs and LHPs, but findings have been inconsistent. One study involving youth baseball pitchers found that RHPs demonstrated significantly greater ER and humeral torsion angle in their throwing arms than in their non-throwing arms [12]. In contrast, LHPs showed no significant side-to-side differences in ER and humeral torsion angle [12]. Similarly, collegiate-level studies found no significant differences in ER or total ROM between RHPs and LHPs, although LHPs demonstrated significantly greater IR in their throwing arms [13]. In professional pitchers, LHPs exhibited lower dominant arm ER and greater limitations in shoulder flexion ROM compared to RHPs [14]. These findings suggest that handedness may influence shoulder and elbow joint adaptations differently across various levels of play. Furthermore, recent findings from high school baseball pitchers demonstrated that LHPs exhibited greater nondominant ER and dominant horizontal adduction (HA) and smaller side-to-side differences in ER, HA, and humeral torsion than those of RHPs [15].

However, existing studies have notable limitations, including small sample sizes and a lack of focus on youth athletes. One study analyzing youth pitchers included only 65 participants (46 RHPs and 19 LHPs) [12], limiting the ability to generalize findings. Moreover, while shoulder ROM and humeral torsion differences between RHPs and LHPs have been explored, potential differences in elbow ROM and shoulder muscle strength remain unclear. Addressing these gaps is essential for establishing a diagnostic baseline for injury risk assessment in LHPs.

This study aimed to compare the biomechanical characteristics of youth RHPs and LHPs by analyzing differences in shoulder and elbow ROM, muscle strength, and humeral torsion angle. By elucidating these handedness-related adaptations, we sought to establish a normative baseline that will facilitate future research on handedness-related adaptations, injury prevention, and performance optimization for both RHPs and LHPs in youth baseball.

## 2. Materials and Methods

### 2.1. Study Design and Participants

This study was a retrospective cross-sectional analysis of youth baseball players who underwent annual preseason medical/physical examinations from 2014 to 2020. Data were collected through standardized orthopedic assessments conducted during these medical checkups. The study population consisted of competitive youth baseball players aged 7–15 years who were actively participating in preseason practice without any restrictions on their pitching or throwing abilities [16]. However, only male players aged 9 to 13 were analyzed, as the sample sizes for female players and male players aged 7, 8, 14, and 15 years were too small for direct comparison with males in other age groups. Additionally, players with only 1–2 years of experience in the sport (aged 7 and 8 years) could not be meaningfully compared to those with 7–8 years of experience (aged 14 and 15 years), further justifying their exclusion. Furthermore, participants were excluded if they had previously sustained an injury to their throwing arm or were unable to play baseball due to shoulder or elbow problems [16]. The study was approved by the Institutional Review Board of Gunma University Hospital (identification number 1003), and all procedures were conducted in accordance with the Declaration of Helsinki and Ethical Guidelines for Medical and Biological Research Involving Human Subjects. Prior to enrollment, written informed consent was obtained from all participants and their parents.

### 2.2. Data Collection

Data were collected and maintained by the staff of the Orthopedic Surgery Department at Gunma University.

#### 2.2.1. Preseason Medical/Physical Examinations

The annual medical/physical examination data were managed and provided by the Department of Orthopedic Surgery of Gunma University as part of its ongoing sports medicine research and preseason screening program for youth baseball players. As in previous studies [17], the current study conducted pre-participation medical/physical examinations in the preseason to establish baseline evaluations of the participants’ shoulder and elbow conditions. To avoid confirmation bias, the examiners were unaware of the participants’ hand dominance. All examinations were conducted under a blinded process, and although the specific examiners varied from year to year, all were orthopedic surgeons who followed the same standardized procedures to ensure consistency and reliability. The reliability of these measurement methods has been validated in our previous studies [16,17,18,19].

We collected information from the participants, coaches, and parents regarding age, sex, and baseball experience, and evaluated the participants’ height, weight, and body mass index. All examinations were performed by orthopedic surgeons. Although the specific examiners varied from year to year, all followed the same standardized procedures to ensure consistency and reliability across seasons.

#### 2.2.2. Shoulder and Elbow ROM

According to previous studies [16,19], the passive ROM in elbow flexion and extension, as well as abduction ER, abduction IR, and HA, of the dominant and nondominant sides were evaluated by a certified orthopedic surgeon using a digital protractor (iGaging, Los Angeles, CA, USA). All measurements were obtained with the participant lying in a supine position. For abduction ER and abduction IR measurement, the examiner applied posterior force to the coracoid process to stabilize the scapula while a second surgeon placed the protractor on the forearm. For HA measurement, the examiner stabilized the axillary border of the scapula while the second certified orthopedic surgeon positioned the protractor on the humerus. For elbow ROM measurement, the fulcrum of the goniometer was positioned over the lateral epicondyle of the humerus, with one arm along the length of the humerus to the tip of the acromion process and the other arm along the length of the radius to the radial styloid process. For each shoulder, the total arc was calculated by adding the ER and IR measurements. The difference in ROM for each measurement was then determined by subtracting the value on the nondominant side from the value on the dominant side.

#### 2.2.3. Shoulder Strength

According to previous studies [16,19], the bilateral shoulder strength of the supraspinatus in prone ER (PER) and prone IR (PIR) were assessed by a certified orthopedic surgeon using a PowerTrack II Commander hand-held dynamometer (J-Tech Medical, Salt Lake City, UT, USA). Shoulder strength was measured in the prone position with the humerus abducted and elbow flexed at 90°. The examiner stabilized the humerus while participants rotated their arm with maximum effort against the dynamometer. For PER strength measurement, the dynamometer was placed on the dorsal side of the forearm, 5 cm proximal to the wrist extension crease. For PIR strength measurement, the dynamometer was placed on the volar side of the forearm, 5 cm proximal to the wrist flexion crease.

Each measurement was repeated three times, and the median value was recorded for analysis. The dominant-to-nondominant ratios of PER and PIR were calculated for each participant and expressed as percentages.

#### 2.2.4. Humeral Torsion

As detailed in prior studies [3], humeral torsion was evaluated using an indirect ultrasound-based technique. All measurements were carried out by a certified orthopedic surgeon with extensive training and experience in orthopedic ultrasound procedures. Based on a previous study [18], the examiner applied a multifrequency 12 MHz linear-array transducer (LOGIQe; GE Healthcare, Chicago, IL, USA) to the anterior shoulder of the participant, positioning it perpendicular to the horizontal plane as verified by a bubble level. The humerus of the participant was then passively rotated until the bicipital groove was centered in the ultrasound image, with the line connecting the peaks of the lesser and greater tubercles aligned parallel to the horizontal plane. Finally, the angle between the forearm and the horizontal plane was measured using a digital inclinometer (Shinwa Rules Co, Sanjo, Niigata, Japan) to determine the humeral retrotorsion angle. Since humeral torsion data were only collected between the 2014 and 2017 seasons, analyses involving this parameter were conducted separately from the full dataset to ensure methodological transparency. Moreover, this data was not used for a logistic regression analysis.

### 2.3. Statistical Analysis

Statistical analyses were performed using IBM SPSS Statistics 29 software (IBM Japan, Ltd., Tokyo, Japan), with a significance threshold of *p* < 0.05. Continuous data are summarized as the mean ± standard deviation. Prior to comparing differences between the dominant and nondominant sides within the LHP and RHP groups, the normality of data distribution was assessed. To ensure appropriate statistical analysis, normality was assessed before conducting comparisons. Parametric tests (*t*-tests) were used for variables that followed a normal distribution, while non-parametric methods (Mann–Whitney U tests) were applied for variables that did not meet normality assumptions. To determine the independent factors associated with being an LHP, logistic regression analysis was performed with explanatory variables that demonstrated significance in the univariate analysis. Multicollinearity among the independent variables was evaluated using variance inflation factors (VIFs), with a value > 3 indicating multicollinearity. Logistic regression analysis was conducted to determine the independent factors associated with being an LHP and to calculate the corresponding odds ratios (ORs) and 95% confidence intervals. Pearson correlation analysis using Prism 8 (GraphPad Software, San Diego, CA, USA) was performed to investigate the relationship between humeral torsion differences and ER/IR differences. All statistical analysis and presentation are consistent with the CHAMP statement [20].

## 3. Results

**Figure 1:**
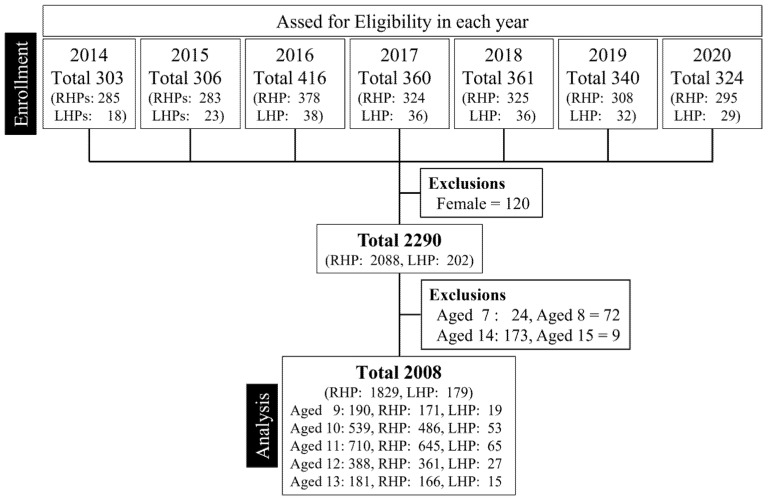
Flow chart of participants enrolled. RHP: right-handed player, LHP: left-handed player.

A total of 2008 male baseball players aged 9–13 years were included in the study, comprising 1829 RHPs (91.1%) and 179 LHPs (8.9%) (Figure 1). Humeral torsion data, collected only between the 2014 and 2017 seasons, totaled 892 measurements from 820 RHPs and 72 LHPs.

The demographic characteristics of the participants are summarized in Table 1. There were no significant differences between the two groups in age, height, weight, body mass index (BMI), or years of baseball experience. The mean age was 10.9 ± 1.1 years for RHPs and 10.8 ± 1.1 years for LHPs. The average height was 143.9 ± 9.7 cm and 142.9 ± 9.9 cm, and the mean body weight was 37.7 ± 9.2 kg and 37.0 ± 8.9 kg for RHPs and LHPs, respectively. The BMI was 17.9 ± 2.9 in RHPs and 17.8 ± 2.9 in LHPs, while baseball experience averaged 3.6 ± 1.5 years and 3.5 ± 1.5 years, respectively.

### 3.1. Comparisons Between LHPs and RHPs

LHPs demonstrated significantly larger nondominant shoulder ER, dominant IR, dominant shoulder HA, and dominant elbow flexion values than those of RHPs. In addition, LHPs demonstrated significantly smaller nondominant shoulder IR, nondominant elbow flexion, dominant PER strength, and nondominant PIR strength values than those of RHPs. No other significant differences were observed between LHPs and RHPs in terms of ROM, shoulder strength, or side-to-side differences in ROM (Table 2).

### 3.2. Side-to-Side Differences (Dominant vs. Nondominant) in LHPs

In LHPs, PIR strength was significantly greater on the dominant side than on the nondominant side, whereas shoulder HA, elbow extension, and PER strength were significantly smaller in their dominant shoulders than in their nondominant shoulders. No other significant differences between the dominant and nondominant sides pertaining to ROM, shoulder strength, or humeral torsion were observed in LHPs (Table 2).

### 3.3. Side-to-Side Differences (Dominant vs. Nondominant) in RHPs

In RHPs, shoulder ER and PER strength were significantly greater in the dominant side than in the nondominant side. In contrast, RHPs exhibited significantly smaller dominant shoulder IR, shoulder HA, elbow extension, and elbow flexion than those of the corresponding nondominant values (Table 2).

### 3.4. Side-to-Side Differences Between RHPs and LHPs

LHPs demonstrated significantly larger side-to-side differences in shoulder IR, HA, elbow flexion, and side-to-side ratio in PIR strength values than those of RHPs. In addition, LHPs demonstrated significantly smaller dominant side-to-side differences in shoulder ER and side-to-side ratio in PER (Table 3).

### 3.5. Comparison of Humeral Torsion Between RHPs and LHPs

In RHPs, humeral torsion was significantly greater in the dominant side than in the nondominant side. Upon comparing RHPs and LHPs, LHPs demonstrated significantly greater side-to-side differences in humeral torsion values than those of RHPs (Table 4).

### 3.6. Correlation Between Humeral Torsion and Shoulder ROM

**Figure 2:**
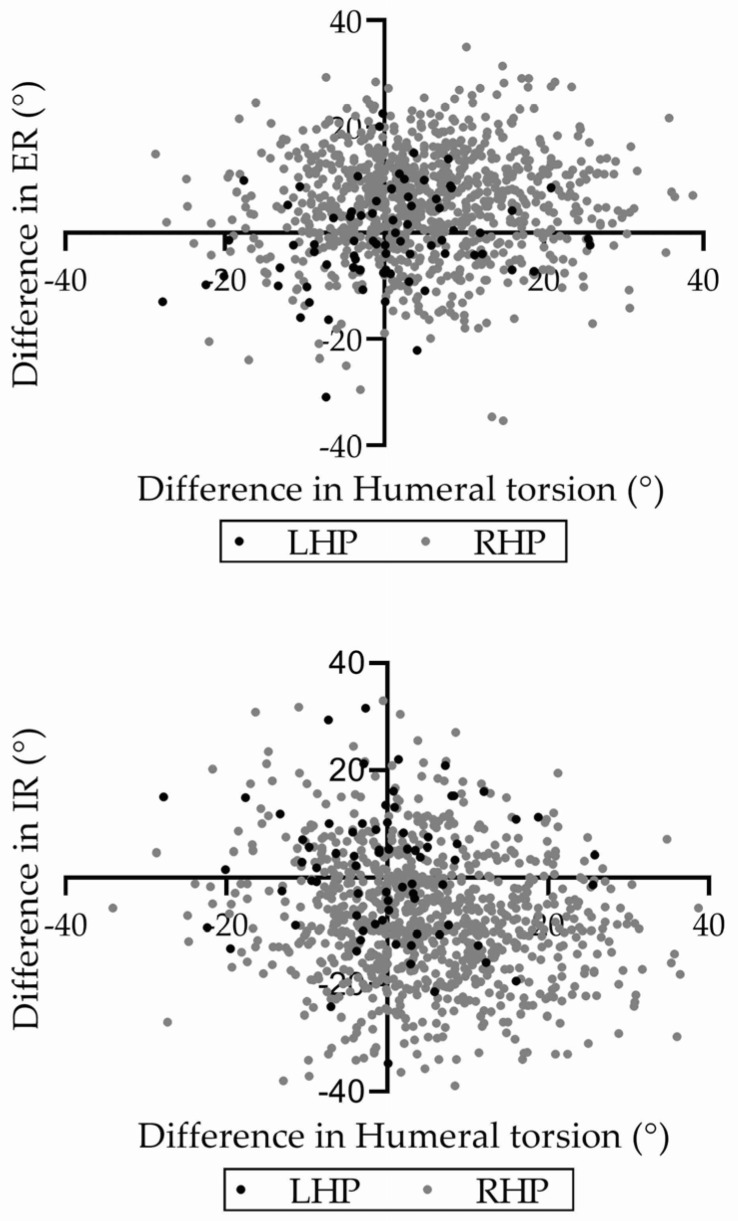
Relationship between humeral torsion difference and shoulder ER/IR differences. The correlation between humeral torsion differences and shoulder external rotation (ER) and internal rotation (IR) differences for LHPs and RHPs are shown. Black dots represent LHPs, and gray dots represent RHPs. Prism 8 (GraphPad Software, San Diego, CA, USA) was utilized to generate these figures.

For the entire cohort, the humeral torsion difference was significantly correlated with the ER difference (r = 0.078; *p* = 0.021) and IR difference (r = −0.160; *p* < 0.0001) (Figure 2). When analyzed by group, the humeral torsion difference was significantly correlated with the IR difference in RHPs (r = −0.151; *p* < 0.0001). However, no significant correlation was observed between the humeral torsion difference and the IR difference in LHPs (r = −0.086; *p* = 0.475) and the ER difference in RHPs (r = 0.054; *p* = 0.126) and in LHPs (r = 0.229; *p* = 0.055).

### 3.7. Logistic Regression Analysis

Univariate analysis identified the following factors associated with being an LHP: nondominant and differences in shoulder ER; dominant, nondominant, and differences in shoulder IR and elbow flexion; dominant and differences in shoulder HA; dominant and nondominant PER; side-to-side ratios in PER and PIR. Differences in nondominant shoulder IR and dominant elbow flexion were removed from the multivariate logistic regression analysis because these differences were calculated for both the dominant and nondominant sides and demonstrated significant correlation (*p* < 0.001). VIFs were calculated for the variables, and nondominant PER (VIF = 20.436) was removed due to multicollinearity. Multivariate logistic regression analysis revealed that a small difference in shoulder ER, large shoulder IR on the dominant side, large difference in shoulder HA, large difference in elbow flexion, small PER ratio, and large PIR ratio were significantly associated with being an LHP (Table 5).

## 4. Discussion

This study found that youth LHPs in our cohort had significantly larger nondominant shoulder ER, dominant IR, dominant shoulder HA, dominant elbow flexion, side-to-side differences in shoulder IR, HA, and elbow flexion, and side-to-side ratio in PIR strength values than those of RHPs. Additionally, LHPs demonstrated significantly smaller values for nondominant shoulder IR, nondominant elbow flexion, dominant PER strength, nondominant PIR strength, side-to-side differences in shoulder ER, and side-to-side ratio in PER strength than those of RHPs. Furthermore, multivariate logistic regression analysis revealed a smaller difference in shoulder ER, greater shoulder IR on the dominant side, larger differences in shoulder HA and elbow flexion, lower PER ratio, and higher PIR ratio as significant factors associated with being an LHP. To the best of our knowledge, this is the first study to comprehensively examine the functional characteristics of LHPs and identify specific physical and anatomical differences in a large sample of youth baseball LHPs and RHPs. By examining not only shoulder ROM and humeral torsion but also elbow ROM and shoulder muscle strength, this study establish an important baseline for identifying unique biomechanical patterns that predispose LHPs to specific injury risks.

### 4.1. Differences in Shoulder and Elbow ROMs Between LHPs and RHPs

Prior research indicates significant differences in shoulder ROM between LHPs and RHPs (Appendix A).

#### 4.1.1. Shoulder ER

Studies have shown that LHPs exhibit significantly smaller dominant shoulder ER [14] than RHPs. However, other studies have found no differences in dominant shoulder ER [12,13,21] between LHPs and RHPs. Consistent with previous studies on youth [12] and college baseball pitchers [12,13,21], the current study revealed no difference in dominant shoulder ER between youth baseball LHPs and RHPs. While some studies have reported significantly greater nondominant shoulder ER in LHPs [14,21] than RHPs, others have indicated no differences in nondominant [12,13] shoulder ER between LHPs and RHPs. Consistent with previous research on professional [18] and college baseball pitchers [21], the current study demonstrated that youth baseball LHPs had significantly greater nondominant shoulder ER than youth baseball RHPs.

In terms of side-to-side differences in shoulder ER, Takeuchi et al. reported that LHPs had significantly smaller differences than RHPs [12]. This was reflected in other studies [13,14,21], wherein the side-to-side differences in shoulder ER in LHPs tended to be smaller than those in RHPs when calculated as the dominant side—nondominant side (Harris et al. [14]: 2.2° vs. 13.9°, Solomito et al. [13]: 10° vs. 17°, and Werner et al. [21]: 0° vs. 13° in LHPs and RHPs, respectively), although not officially reported (Appendix A). Consistent with studies on youth [12], collegiate [13,21], and professional [14] pitchers, the current study demonstrated significantly smaller side-to-side differences in shoulder ER in LHPs than in RHPs.

These findings suggest that LHPs exhibit a more symmetrical shoulder ER profile, with less pronounced adaptation in the dominant arm compared to RHPs. In contrast, RHPs showed significantly larger side-to-side differences in ER, indicating greater dominant-side adaptation likely due to repetitive throwing. This asymmetry may reflect cumulative mechanical loading differences, as RHPs are more common and potentially exposed to higher competitive demands and training volumes from earlier stages in youth development. The more balanced ER in LHPs may suggest a different neuromuscular or biomechanical adaptation pathway, potentially offering protective benefits against shoulder injuries associated with excessive ER gain. Conversely, the greater ER asymmetry in RHPs might increase their risk for posterior capsule tightness, internal impingement, or other shoulder pathologies [22,23].

#### 4.1.2. Shoulder IR

LHPs have been shown to exhibit significantly greater dominant shoulder IR [13,14] and smaller nondominant shoulder IR [14] than RHPs. However, some studies have found no differences in dominant [12,21] and nondominant [12,13,21] shoulder IR between LHPs and RHPs. Consistent with previous research on professional baseball pitchers [14], the current study also demonstrated that LHPs had significantly greater dominant shoulder IR and smaller nondominant shoulder IR than RHPs among youth baseball players.

Previous studies have shown that no differences in the dominant and nondominant shoulder total arc between LHPs and RHPs in professional [14] and collegiate [13] pitchers. Consistent with those studies, the present study also found no significant difference in dominant shoulder total arc between LHPs and RHPs. 

In terms of side-to-side differences in shoulder IR, Harris et al. found that LHPs had significantly smaller side-to-side differences in shoulder IR than RHPs [14]. Although the other studies did not report side-to-side differences in IR, the values tended to be smaller for LHPs than for RHP when calculated as the dominant side—nondominant side (Takeuchi et al. [12]: −7° vs. −8°, Solomito et al. [13]: −2° vs. −13°, and Werner et al. [21]: −3° vs. −11° in LHPs and RHPs, respectively).

Similar to shoulder ER, the sample size may have impacted the statistical significance of these findings. In line with prior research on youth [12], collegiate [13,21], and professional [14] players, the current study demonstrates that side-to-side differences in shoulder IR were significantly smaller in LHPs than in RHPs. Moreover, shoulder IR also demonstrated less side-to-side asymmetry in LHPs compared to RHPs across all levels of play. This suggests that RHPs may experience greater adaptive stress on the shoulder joint, potentially contributing to asymmetrical IR patterns and an elevated risk for overuse injuries such as glenohumeral internal rotation deficit (GIRD). These findings reinforce the need for handedness-specific assessment and prevention strategies in youth baseball, particularly for RHPs who may be more prone to developing maladaptive shoulder adaptations.

#### 4.1.3. Shoulder HA

The current study revealed that LHPs had significantly greater dominant shoulder HA than RHPs, no differences in nondominant shoulder HA between LHPs and RHPs, and significantly smaller side-to-side differences in shoulder HA than RHPs. This pattern, similar to that seen in shoulder ER and IR, suggests that LHPs demonstrate less asymmetry in shoulder HA. Previous biomechanical studies have shown that LHPs exhibit smaller shoulder HA angles and lower HA angular velocity during the pitching motion—from foot contact to ball release—compared with RHPs [13,21], indicating a different orientation and alignment of the throwing arm. Although LHPs have greater static HA ROM, their dynamic HA motion and angular velocity are smaller, which may contribute to reduced shoulder distraction stress during pitching [24]. Thus, our findings suggest that LHPs may experience less mechanical stress on the shoulder despite having greater static HA flexibility.

Taken together with the ER and IR findings, the reduced HA asymmetry observed in LHPs supports the hypothesis of handedness-related biomechanical adaptations. These more symmetrical shoulder movement profiles may contribute to a reduced risk of overuse injuries in LHPs. However, further studies are needed to validate these interpretations and investigate their long-term clinical significance in throwing athletes.

#### 4.1.4. Elbow ROM

The current study revealed that LHPs exhibited significantly greater dominant-side elbow flexion, smaller nondominant-side elbow flexion, and smaller side-to-side differences in elbow flexion compared with RHPs. In contrast, no significant differences were observed between LHPs and RHPs in terms of elbow extension on either side or side-to-side differences in elbow extension. This pattern is similar to the differences observed in shoulder IR, suggesting that LHPs tend to maintain a more symmetrical joint mobility profile across the upper extremities.

Previous biomechanical studies have shown that repetitive throwing increases dominant-side elbow flexion in youth pitchers due to soft-tissue adaptation [25], and that greater elbow flexion during pitching can reduce medial elbow valgus torque [26]. Furthermore, Solomito et al. [13] reported that LHPs demonstrate greater elbow flexion at foot contact during pitching compared with RHPs, possibly reflecting distinct throwing kinematics. These findings collectively support the present results and suggest that the elbow flexion characteristics observed in LHPs may represent adaptive responses that reduce stress on the medial elbow structures.

The reduced asymmetry in elbow flexion observed in LHPs may indicate a lower degree of repetitive stress concentration on the medial elbow structures. This, in turn, could potentially reduce their susceptibility to valgus extension overload and medial elbow injuries, such as ulnar collateral ligament insufficiency. Future studies integrating biomechanical modeling and longitudinal injury surveillance will be valuable in clarifying the clinical relevance of these elbow ROM differences.

The present study demonstrates that LHPs exhibit more symmetrical joint mobility profiles across multiple upper extremity joints—including the shoulder ER, IR, HA, and elbow flexion—compared with RHPs. These differences were consistently observed as smaller side-to-side asymmetries in LHPs, supporting the notion of handedness-specific biomechanical adaptations. While RHPs exhibited greater side-to-side differences, likely reflecting dominant-side adaptations due to higher mechanical loads and earlier specialization, LHPs maintained a more balanced pattern of mobility that may confer a protective advantage against overuse injuries. These findings highlight the importance of assessing bilateral joint function and considering handedness in both performance evaluation and injury prevention strategies for youth baseball players. Further longitudinal studies are warranted to explore how these mobility characteristics evolve with age and training exposure and to clarify their role in long-term musculoskeletal health.

### 4.2. Differences in Shoulder Strength Between LHPs and RHPs

In the current study, LHPs exhibited significantly lower dominant-side PER strength and lower nondominant-side PIR strength compared to RHPs. Additionally, LHPs demonstrated a significantly lower side-to-side ratio in PER strength and a higher ratio in PIR strength. These findings suggest a tendency in LHPs toward greater internal rotation dominance relative to external rotation strength. Previous biomechanical studies have shown that LHPs exhibit lower maximum shoulder IR torque during the pitching motion compared to RHPs at the collegiate level [21], which aligns with our findings. However, other studies in professional pitchers revealed no such differences [13], potentially due to advanced neuromuscular adaptation in elite-level athletes. The asymmetrical shoulder strength profile in RHPs may reflect more pronounced training-induced adaptations or compensatory mechanisms due to repetitive high-volume throwing. In contrast, LHPs may develop more balanced strength distributions or exhibit different recruitment patterns of rotator cuff muscles such as the infraspinatus, teres minor, subscapularis, and pectoralis major.

### 4.3. Differences in Humeral Torsion Between LHPs and RHPs

Previous studies have revealed results similar to those of this study regarding humeral torsion differences between LHPs and RHPs (Appendix B). Consistent with these prior studies, the current results revealed notable differences in humeral torsion between LHPs and RHPs, particularly in side-to-side asymmetry. Regarding the dominant side, Takeuchi et al. reported that youth LHPs exhibited significantly smaller humeral torsion angles compared to RHPs [12]. In contrast, Harris et al. found no significant difference in professional pitchers, although LHPs tended to have lower values [14]. Our findings align with the latter, showing no statistically significant difference in dominant-side humeral torsion between groups. However, LHPs demonstrated a trend toward smaller angles, supporting the notion of handedness-related differences in adaptation.

For the nondominant side, Harris et al. demonstrated that professional LHPs had significantly greater humeral torsion than RHPs [14], while Takeuchi et al. reported no significant difference in youth athletes [12]. Similarly, the current study revealed no significant difference in nondominant humeral torsion between youth LHPs and RHPs; however, a trend toward greater torsion in LHPs was observed.

Regarding side-to-side differences, previous studies have consistently shown that LHPs exhibit significantly smaller humeral torsion asymmetry than RHPs [12,14]. The current findings corroborate these observations, with LHPs showing reduced dominant-side torsion and significantly smaller side-to-side differences.

These patterns suggest that RHPs may undergo more pronounced bony remodeling in response to repetitive throwing loads, while LHPs exhibit less adaptation, potentially due to differences in cumulative throwing volume, mechanical stress distribution, or early training exposure. Because RHPs generally outnumber LHPs in youth baseball, RHPs often experience greater cumulative pitching exposure and competitive demands throughout early development, which may accelerate osseous adaptation. This interpretation aligns with the established concept of humeral osseous remodeling resulting from repetitive throwing [27] and is consistent with previous comparative findings showing greater humeral retrotorsion in RHPs [16,18]. Together, these observations provide a plausible explanation for the asymmetric skeletal adaptation patterns observed in this study. Given the established relationship between humeral torsion and shoulder ROM, our findings reinforce the hypothesis that handedness influences skeletal adaptation patterns in youth baseball players. This underscores the importance of individualized assessment strategies that consider the structural and functional differences associated with throwing arm dominance.

Previous research on professional pitchers established a positive correlation between increased humeral torsion and greater shoulder ER [14,27,28]. The current study corroborates these previous findings [14,27,28], demonstrating that decreased humeral retrotorsion in LHPs, compared with that in RHPs, is associated with reduced shoulder ER in LHPs relative to RHPs. Furthermore, previous studies found associations between increased humeral retrotorsion and increased IR (GIRD) [14,29]. Consistent with these studies, the current study also demonstrated LHPs exhibited significantly lower humeral torsion and GIRD than RHPs.

### 4.4. Independent Factors Associated with Being an LHP

The current study demonstrated that increased dominant shoulder IR, side-to-side difference in shoulder HA, side-to-side difference in elbow flexion, and PIR strength and decreased side-to-side difference in shoulder ER and side-to-side ratios in PER were significantly associated with being an LHP. To interpret the effects of each independent factor associated with being an LHP, we calculated the ORs of the conditions based on the mean differences between groups. If side-to-side differences in shoulder ER decreased by 6.6°, dominant shoulder IR increased by 2.6°, side-to-side differences in shoulder HA increased by 3.6°, side-to-side difference in elbow flexion increased by 2.0°, side-to-side ratios in PER decreased by 10.4%, and PIR strength increased by 7.3%, and the likelihood of the player being an LHP increased by 45%, 5%, 16%, 21%, 43%, and 15%, respectively (Appendix C). Among these, two factors stood out: a decreased side-to-side difference in shoulder ER and a lower side-to-side ratio in PER strength. Both of these variables showed the highest calculated ORs—approximately 45% each—for predicting LHP status when comparing mean differences between groups. This suggests that limited ER asymmetry and reduced dominant-side PER strength may be hallmark features of LHPs in youth baseball.

### 4.5. Possible Etiology of Handedness-Related Differences

The underlying mechanisms driving the differences between left- and right-handed pitchers remain incompletely understood. Current evidence suggests that handedness-related asymmetry results from the interaction between innate neurodevelopmental lateralization and acquired structural adaptation.

From a neurodevelopmental perspective, handedness originates from early asymmetry in the spinal cord and motor cortical circuits that promote lateralized control of limb movement [30,31,32]. This neural lateralization facilitates dominant-side motor efficiency and preferential use from early development, leading to asymmetric mechanical loading during growth. Over time, repetitive throwing on the dominant side induces structural remodeling—including increased humeral retrotorsion [33] and capsular or muscular adaptation [34]—that modifies shoulder range of motion and internal/external rotation balance.

### 4.6. Limitations

While our study provides valuable insights, it is limited by its cross-sectional design, which prevents causal inferences. Additionally, although our large sample size strengthens the reliability of our findings, the wide age range of participants (9–13 years) and varying levels of baseball experience may introduce developmental differences that could influence shoulder and elbow biomechanics. Future longitudinal studies are needed to assess how growth, training adaptations, and competition level affect these biomechanical characteristics over time.

Moreover, previous studies investigated the differences between LHPs and RHPs included only pitchers, making it difficult to compare them to the participants in the current study, which included field players in addition to pitchers. However, in the youth age group, the demands specific to the pitcher position may not be as pronounced as at professional or collegiate level because they often play multiple positions.

A further limitation is the imbalance in sample size between right-handed and left-handed players. Because left-handed players naturally constitute a small proportion of the baseball population, the number of left-handed participants (*n* = 179) was relatively limited compared with the right-handed group (*n* = 1829). This inherent imbalance may have influenced the statistical power and precision of between-group comparisons, and the results should therefore be interpreted with caution.

In addition, the present study included only male youth baseball players aged 9–13 years; therefore, the findings may not be generalizable to female athletes or to other age groups. Furthermore, information regarding field practice load, participation in other sports, and other extrinsic or intrinsic factors that could have influenced the results was not collected in detail. These unmeasured variables may have affected the biomechanical characteristics observed in this study. Lastly, the focus on youth baseball players may limit the generalizability of our findings to other competitive levels. Hence, further longitudinal investigations could elucidate the long-term performance and injury implications of the observed biomechanical differences. Future research should also seek to uncover the underlying mechanisms driving these adaptations and explore their relevance for training and rehabilitation approaches.

## 5. Conclusions

This study provides a comprehensive biomechanical assessment of LHPs, highlighting key differences from RHPs in youth male baseball players aged 9–13 years. LHPs exhibited greater nondominant shoulder ER, dominant shoulder IR, and dominant elbow flexion, and lower dominant shoulder ER and smaller side-to-side differences in shoulder ROM and humeral torsion. These findings suggest that LHPs develop more symmetrical adaptations compared to RHPs. Additionally, LHPs demonstrated distinct shoulder strength patterns, with lower side-to-side PER ratios and higher PIR ratios, reflecting handedness-specific muscle adaptations. Logistic regression analysis identified dominant shoulder ER, nondominant shoulder ER, dominant shoulder IR, and strength asymmetries as key factors distinguishing LHPs from RHPs. By establishing a diagnostic baseline for LHPs, this study fills a critical gap in the literature and provides valuable insights for injury prevention, training strategies, and performance optimization in youth baseball players.

## Figures and Tables

**Table 1:** Demographic characteristics of right-handed and left-handed players.

	RHPs	LHPs	*p*-Value
	(*n* = 1829)	(*n* = 179)	
Age (years)	10.9 ± 1.1	10.8 ± 1.1	0.175
Height (cm)	143.9 ± 9.7	142.9 ± 9.9	0.211
Weight (kg)	37.7 ± 9.2	37.0 ± 8.9	0.298
BMI	17.9 ± 2.9	17.8 ± 2.9	0.572
Baseball experience (years)	3.6 ± 1.5	3.5 ± 1.5	0.512

Continuous data are expressed as the mean ± SD. RHP: right-handed player, LHP: left-handed player, BMI: body mass index.

**Table 2:** Differences in shoulder and elbow ROM and shoulder strength: within-group comparisons (dominant vs. nondominant) and between-group comparisons (RHPs vs. LHPs).

		RHPs (*n* = 1829)	Dom vs. NDom Within RHPs	LHPs (*n* = 179)	Dom vs. NDom Within LHPs	RHPs vs. LHPs
			*p*-Values		*p*-Values	*p*-Values
Shoulder ROM (°)						
ER	Dom	115.4 ± 10.2	<0.0001 *	113.8 ± 11.7	0.563	0.079
	N Dom	109.3 ± 11.6		114.3 ± 10.9		<0.001 #
IR	Dom	44.4 ± 12.0	<0.0001 *	47.0 ± 11.7	0.522	0.006 #
	N Dom	50.8 ± 13.0		47.9 ± 12.5		0.004 #
HA	Dom	16.2 ± 10.0	<0.0001 *	18.3 ± 10.2	<0.0001 *	0.01 #
	N Dom	24.1 ± 12.7		22.6 ± 12.2		0.126
Total arc	Dom	159.8 ± 15.6	0.238	160.8 ± 16.6	0.162	0.427
	N Dom	160.0 ± 16.8		162.1 ± 17.1		0.11
Elbow ROM (°)						
Extension	Dom	6.5 ± 5.4	<0.0001 *	6.7 ± 5.4	<0.0001 *	0.547
	N Dom	7.0 ± 5.4		7.3 ± 5.5		0.419
Flexion	Dom	140.5 ± 5.8	<0.0001 *	141.6 ± 5.5	0.504	0.018 #
	N Dom	142.3 ± 5.3		141.4 ± 5.2		0.025 #
Shoulder strength (kgf)						
PER	Dom	7.8 ± 2.1	<0.0001 *	7.2 ± 2.1	0.005 *	0.004 #
	Ndom	7.4 ± 2.1		7.7 ± 2.2		0.209
PIR	Dom	8.5 ± 2.4	0.416	8.6 ± 2.7	<0.0001 *	0.767
	Ndom	8.5 ± 2.5		7.9 ± 2.3		0.013 #

Data are expressed as the mean ± SD for the dominant throwing arm (Dom) and nondominant (NDom) arm. ER: external rotation, IR: internal rotation, HA: humeral torsion, Total arc is defined as the shoulder ER plus IR value. PER/PIR: prone external/internal rotation, SS: seated supraspinatus, ratio (%) is defined as the Dom divided by the NDom arm value multiplied 100. “Dom vs. NDom” refers to the comparison within subjects, specifically between the dominant and nondominant sides within each group (RHPs and LHPs) using Wilcoxon Signed-Rank test (* Statistically significant). “RHPs vs. LHPs” refers to the comparison between subjects, assessing differences in the dominant and nondominant sides between RHPs and LHPs using Mann–Whitney U test (# Statistically significant).

**Table 3:** Comparison of side-to-side differences in shoulder and elbow ROM and shoulder strength between right-handed and left-handed players.

	RHPs	LHPs	*p*-Values
	(*n* = 1829)	(*n* = 179)	
Side-to-side differences of shoulder ROM (°)			
ER	6.1 ± 10.6	−0.5 ± 8.9	<0.001 #
IR	−6.4 ± 12.3	−0.9 ± 13.1	<0.001 #
HA	−7.8 ± 11.1	−4.3 ± 10.8	<0.001 #
Total arc	−0.2 ± 14.4	−1.4 ± 15.2	0.32
Side-to-side differences of elbow ROM (°)			
Extension	−0.5 ± 3.6	−0.6 ± 4.8	0.818
Flexion	−1.8 ± 4.7	0.2 ± 4.4	<0.001 #
Side-to-side ratios of shoulder strength (%)			
PER	106.8 ± 20.9	96.4 ± 18.6	<0.001 #
PIR	102.5 ± 20.0	109.8 ± 22.5	<0.001 #

Data are expressed as the mean ± SD for the dominant throwing arm (Dom) and nondominant (NDom) arm. ER: external rotation, IR: internal rotation, HA: humeral torsion, Total arc is defined as the shoulder ER plus IR value. PER/PIR: prone external/internal rotation, ratio (%) is defined as the Dom divided by the NDom arm value multiplied 100, # Statistically significant.

**Table 4:** Differences in humeral torsion: within-group comparisons (dominant vs. nondominant) and between-group comparisons (RHPs vs. LHPs).

		RHPs (*n* = 820)	Dom vs. NDom Within RHPs	LHPs (*n* = 72)	Dom vs. NDom Within LHPs	RHPs vs. LHPs
			*p*-Values		*p*-Values	*p*-Values
Humeral torsion (°)	Dom	75.6 ± 9.7	<0.0001 *	73.7 ± 10.4	0.806	0.121
	NDom	71.3 ± 11.4		73.0 ± 10.3		0.239
	Diff	4.2 ± 10.8		0.4 ± 9.9		0.004 #

Data are expressed in the mean ± SD for the dominant throwing arm (Dom) and nondominant (NDom) arm. Dif: Difference is defined as the Dom minus the NDom arm value. “Dom vs. NDom” refers to the comparison within subjects, specifically between the dominant and nondominant sides within each group (RHPs and LHPs) using Wilcoxon Signed-Rank test (* Statistically significant). “RHPs vs. LHPs” refers to the comparison between subjects, assessing differences in the dominant side, nondominant side, and side-to-side difference between RHPs and LHPs using Mann–Whitney U test (# Statistically significant).

**Table 5:** Results of the logistic regression analysis.

	Odds Ratio	95% CI	*p*-Value	VIF
Shoulder ER in the NDom (°)	1.013	0.992–1.034	0.225	1.639
Difference in shoulder ER (°)	0.945	0.925–0.967	<0.001 *	1.612
Shoulder IR in the Dom (°)	1.02	1.002–1.038	0.034 *	1.413
Difference in shoulder IR (°)	1.016	0.998–1.033	0.079	1.395
Shoulder HA in the Dom (°)	1.011	0.991–1.031	0.272	1.229
Difference in shoulder HA (°)	1.042	1.022–1.063	<0.001 *	1.175
Elbow flexion in the NDom (°)	0.995	0.955–1.036	0.800	1.186
Difference in elbow flexion (°)	1.101	1.055–1.15	<0.001 *	1.163
PER in the Dom (kgf)	1.028	0.929–1.137	0.598	1.175
PER ratio (%)	0.966	0.955–0.977	<0.001 *	1.110
PIR ratio (%)	1.019	1.010–1.028	<0.001 *	1.020

CI: confidence interval, VIF: variance inflation factor, Dom: dominant side, NDom: nondominant side, ER/IR: external/internal rotation, HA: horizontal adduction, PER/IR: prone external/internal rotation strength, ratio (%) is defined as the Dom divided by the NDom arm value multiplied 100, Difference is defined as the Dom minus the NDom arm value, * Statistically significant.

## Data Availability

The data supporting the findings of this study are available from the corresponding author (H.S.) upon request. The data are not publicly available because they contain information that could compromise the privacy of the participants.

## Abbreviations

The following abbreviations are used in this manuscript:

Dom	Dominant
ER	External rotation
HA	Horizontal adduction
IR	Internal rotation
LHPs	Left-handed players
NDom	Nondominant
OR	Odds ratio
PER	Prone external rotation
PIR	Prone internal rotation
RHPs	Right-handed players
ROM	Range of motion
VIFs	Variance initiation factors

## Appendix A

**Table A1:** Comparison of Current Results and Previous Studies in Shoulder ROM.

		Current Study			Takeuchi et al., 2019 [12]			Harris et al., 2022 [14]			Solomito et al., 2017 [13]			Werner et al., 2010 [21]		
	Arm	RH Player	LH Player	*p*-Value	RH Pitcher	LH Pitcher	*p*-Value	RH Pitcher	LH Pitcher	*p*-Value	RH Pitcher	LH Pitcher	*p*-Value	RH Pitcher	LH Pitcher	*p*-Value
Level		Youth Aged 9 to 13 Years			Youth Aged 9 to 12 Years			Professional			Collegiate			Collegiate		
Sample size		1829	179		46	19		160	57		26	26		28	28	
ER (°)	Dom	115.4± 10.2	113.8 ± 11.7	0.079	116 ± 14	113 ± 11	0.394	118.5 ± 8.8	112.7 ± 9.1	<0.001 *	122 ± 19	122 ± 17	0.977	126 ± 12	124 ± 11	-
	NDom	109.3 ± 11.6	114.3 ± 10.9	<0.001 *	111 ± 13	114 ± 12	0.312	104.6 ± 8.3	110.5 ± 8.9	<0.001 *	105 ± 17	112 ± 27	0.311	113 ± 9	124 ± 8	<0.001 *
	Diff	6.1 ± 10.6	−0.5 ± 8.9	<0.001 *	5 ± 8	−2 ± 8	<0.001 *									
IR (°)	Dom	44.4 ± 12.0	47.0 ± 11.7	0.006 *	41 ± 15	41 ± 17	0.956	45.9 ± 7.2	51.0 ± 7.9	<0.001 *	44 ± 14	53 ± 17	0.029 *	44 ± 8	19 ± 10	-
	NDom	50.8 ± 13.0	47.9 ± 12.5	0.004 *	49 ± 16	48 ± 16	0.888	59.8 ± 7.6	55.8 ± 6.9	0.001 *	57 ± 15	55 ± 15	0.734	55 ± 9	52 ± 12	-
	Diff	−6.4 ± 12.3	−0.9 ± 13.1	<0.001 *				−13.9 ± 6.3	−4.8 ± 6.4	<0.001 *						
Total arc (°)	Dom	159.8 ± 15.6	160.0 ±16.6	0.427				164.5 ± 10.8	163.6 ± 10.2	0.591	165 ± 22	175 ± 22	0.281			
	NDom	160.0 ± 16.8	162.1 ± 17.1	0.110				164.3 ± 9.7	163.3 ± 9.9	0.186	169 ± 26	170 ± 27	0.928			
	Diff	−0.2 ± 14.4	−1.4 ± 15.2	0.320				0.0 ± 7.2	2.8 ± 9.0	0.002 *						

Continuous data are reported as means ± standard deviations. RH, right-handed; LH, left-handed; Dom, dominant arm; NDom, nondominant arm; ER, external rotation; IR, internal rotation; Total arc is defined as the sum of ER and IR values; Diff, Difference is defined as the Dom minus the NDom arm value; * Statistically significant.

## Appendix B

**Table A2:** Comparison Between Current Results and Those in Previous Studies on Humeral Torsion.

		Current Study			Takeuchi et al., 2019 [12]			Harris et al., 2022 [14]		
	Arm	RH Player	LH Player	*p*-Value	RH Pitcher	LH Pitcher	*p*-Value	RH Pitcher	LH Pitcher	*p*-Value
Level		Youth Aged 9 to 13 Years			Youth Aged 9 to 12 Years			Professional		
Sample size		820	72		46	19		160	57	
Humeral torsion	Dom	75.6 ± 9.7	73.7 ± 10.4	0.121	84 ± 9	79 ± 10	0.033 *	92.3 ± 13.0	81.4 ± 8.5	0.120
	NDom	71.3 ± 11.4	73.0 ± 10.3	0.239	77 ± 9	77 ± 9	0.991	69.2 ± 12.4	79.3 ± 8.5	<0.001 *
	Diff	4.2 ± 10.8	0.4 ± 9.9	0.004 *	8 ± 7	2 ± 5	<0.001 *	23.1 ± 10.9	2.2 ± 5.8	<0.001 *

Continuous data are reported as means ± standard deviations. RH, right-handed; LH, left-handed; Dom, dominant arm; NDom, nondominant arm; Diff, Difference is defined as the Dom minus the NDom arm value; * Statistically significant.

## Appendix C

**Table A3:** Odds Ratios for Factors Associated with Being a Left-Handed Player.

	ORs	Mean	Calculated
		Difference	ORs
Diff in shoulder ER (°)	0.945	−6.6	1.453
Shoulder IR in the Dom (°)	1.020	2.6	1.052
Diff in shoulder HA (°)	1.042	3.6	1.157
Diff in elbow flexion (°)	1.101	2.0	1.214
PER ratio (%)	0.966	−10.4	1.434
PIR ratio (%)	1.019	7.3	1.148

Diff: Difference is defined as the Dom minus the NDom arm value. Ratio (%) is defined as the Dom divided by the NDom arm value multiplied 100, Mean difference is defined as the LHPs minus the RHPs value. Dom, dominant side; ER/IR, external/internal rotation; HA, horizontal adduction; PER/IR, prone external/internal rotation strength; OR, odds ratio.
